# The rapid kinetics of optimal treatment with subcutaneous methotrexate in early inflammatory arthritis: an observational study

**DOI:** 10.1186/s12891-016-1213-6

**Published:** 2016-08-24

**Authors:** Anna O’Connor, Carter Thorne, Hyeon Kang, Diane Tin, Janet E. Pope

**Affiliations:** 1 Schulich School of Medicine & Dentistry, University of Western Ontario, 268 Grosvenor St, London, ON N6A 4V2 Canada; 2 Southlake Regional Health Centre, Newmarket, Canada; 3 University of Toronto, Toronto, ON Canada; 4 Rheumatology, St. Joseph’s Health Care, London, ON Canada

**Keywords:** ERA (early RA), Methotrexate, Optimal treatment strategy, Subcutaneous (sc)

## Abstract

**Background:**

Methotrexate (MTX) is standard treatment for RA. Absorption is better in subcutaneous MTX (scMTX), which may impact speed of onset. In RA, earlier time to remission improves long-term results. Our objectives were to determine rapidity of response of subcutaneous methotrexate in early rheumatoid arthritis.

**Methods:**

The change in several disease activity measures (including DAS28) from 0 to 6 weeks (early period) and 6 to 12 weeks (late period) was compared. The proportion achieving DAS28/CDAI/SDAI remission and/or low disease activity state was also compared.

**Results:**

One hundred three patients were included from a single site between 2008 and 2014. All received MTX (98.0 % scMTX, 98 % 25 mg/week). There were no dropouts. There was a significantly greater early change in DAS28 (−1.9 vs. −0.2, *p* < 0.00); this effect was seen for several outcome measures. By 6 weeks, 59 % had achieved either DAS28 remission or low disease activity state, with 74 % achieving either state by 12 weeks.

There were a larger proportion of patients achieving CDAI and DAS28 remission in the early versus late period (*p* < 0.0002 for both). There was significant improvement when using combination MTX and HCQ, however sample size was small (*n* = 9). The use of intra-articular steroids with MTX yielded the most disease measures that demonstrated early significant improvement.

**Conclusion:**

Subcutaneous MTX is rapid, as the change in many disease activity scores was significantly greater between 0–6 weeks compared to 6–12 weeks. Combination MTX + HCQ gave added value, although generalizability is limited by combination cohort sample size. Intra-articular steroid injections may contribute to the early effect.

**Electronic supplementary material:**

The online version of this article (doi:10.1186/s12891-016-1213-6) contains supplementary material, which is available to authorized users.

## Background

Rheumatoid arthritis (RA) is a chronic autoimmune inflammatory disease characterized by joint swelling, pain that can lead to joint damage, functional impairment, disability, deterioration of quality of life, and premature mortality [1–3]. Initiating treatment for RA early in the disease process can lead to improved long-term outcomes, suggesting that there is an optimal window of opportunity for achieving treatment targets [2, 4–6].

Despite the existence of newer anti-rheumatic drugs (disease modifying anti-rheumatic drugs; DMARDs), methotrexate (MTX) remains the most widely used DMARD because it is effective, has an acceptable toxicity profile, and inexpensive [7–11]. Dosing and route of MTX administration have evolved. Early trials started with low doses (5–7.5 mg/week); however, modern practice typically involves starting at or rapidly escalating to higher doses (15–25 mg/week) for optimal outcomes [12–15]. Route of MTX delivery may also impact disease outcomes; bioavailability is higher when given subcutaneously (sc), possibly relating to limitations in gastrointestinal tract absorption and patient adherence secondary to GI side effects [12, 13]. A previous study of oral and sc MTX in patients with RA suggested that oral administration was less effective [12]. A separate observational study demonstrated a significant association between initial sc administration of MTX and treatment continuation over the first year compared to oral MTX in patients with early RA [13].

The kinetics of optimal initial dosing with subcutaneous methotrexate are unclear. Methotrexate has traditionally been considered slow acting, especially in comparison to newer biologic therapies, with recommendations to reassess clinical response to MTX at 12 weeks [7]. However, this has not been our clinical impression with parenteral MTX. The objective of this study was to determine the kinetics of optimizing initial dosing with sc MTX in patients with early rheumatoid arthritis, comparing outcomes at 6 and 12 weeks after initiation of methotrexate with or without other treatment such as intra-articular steroids.

## Methods

### Study design

Data were collected retrospectively from a single site inception cohort of patients enrolled into the Canadian Early Arthritis Cohort (CATCH) study. CATCH is an observational, prospective “real world” cohort of patients with early inflammatory arthritis (EIA) recruiting at 15 sites since July 2007. Inclusion criteria were: age >16 years, between 6 weeks and 12 months of persistent synovitis at time of entry, and ≥ 2 swollen joints or 1 swollen metacarpophalangeal or proximal interphalangeal joint and ≥ 1 of the following were required: positive rheumatoid factor (RF), positive anti-cyclic citrullinated peptide (anti-CCP), morning stiffness >45 min, response to nonsteroidal anti-inflammatory drugs, or painful metatarsophalangeal squeeze test. Outcomes are collected at each visit including global assessments, joint counts, and inflammatory markers. Treatment was left to the discretion of the treating physician and included: monotherapy or combination DMARD therapy, steroids, NSAIDs and biologics. CATCH patients enrolled at a single site (Southlake Regional Health Centre) were included if they: attended an additional “early follow-up” appointment 6 ± 2 weeks from baseline, attended a 12-week follow up appointment, were prescribed methotrexate within 3 months from their baseline appointment, and were on methotrexate at least 1 month between baseline and the 6 ± 2 week follow-up appointment. At early follow-up, the standard CATCH protocol was used to evaluate patients. Data from early follow-up were collected from paper and/or electronic records as this visit was not part of the standard CATCH protocol. One individual (HK) was responsible for data collection and coding.

Disease measures assessed at baseline, 6 and 12 weeks included: swollen joint count (SJC), 28-tender joint count (TJC28), 28-swollen joint count (SJC28), 28-joint disease activity score (DAS28ESR, DAS28CRP), sleep, pain, fatigue, physician global assessment, patient global assessment, health assessment questionnaire (HAQ), ESR, and CRP. Other composite scores were calculated, including the clinical disease activity index (CDAI) and simplified disease activity index (SDAI). Data regarding tolerability to methotrexate were also collected, including incidence of headache, nausea, and gastrointestinal side effects.

### Statistical methods

A repeated measures ANOVA was performed to compare each disease activity score at baseline, 6 and 12 weeks. A repeated measures ANOVA was also performed on the calculated mean difference in disease activity scores between baseline and 6 weeks, 6 and 12 weeks, and baseline and 12 weeks. A paired *t*-test was performed to compare the mean difference in disease activity scores between early (baseline to 6 weeks) and late (6–12 weeks) groups.

Subgroup analyses assessed the effect of confounding concomitant steroid use and combination therapy with HCQ. Patients were classified according to route of steroid use: intra-articular, oral, intra-muscular, combination, and no steroid. A repeated-measures ANOVA compared raw disease activity scores and the mean difference between time points. Post hoc analysis compared mean scores between early (baseline to 6 weeks) and late (6–12 weeks) groups across routes of steroid use. A similar analysis was performed to compare scores in patients receiving monotherapy versus combination therapy with HCQ.

## Results

### Patient population

**Fig. 1:**
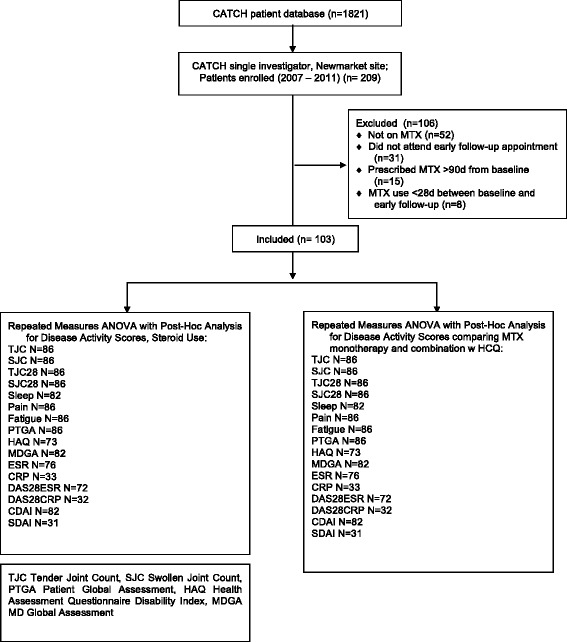
Patient disposition

A total of 103 of 209 CATCH patients enrolled at the single site were included. The mean age was 56.2 years (SD 16.0), with 64 % female (Fig. 1 and Table 1). Almost all (98 %) were on subcutaneous methotrexate, and 2 % on oral methotrexate. Mean symptom duration at first visit was 159 days (SD 106), and 83 % met 2010 ACR/EULAR criteria for rheumatoid arthritis. More than half (59 %) were rheumatoid factor positive. Majority (57 %) were ever-smokers, 85 % Caucasian, 61 % married, and 56 % had post-secondary education. Half were employed, and 54 % had an income between $20,000 and $100,000. The mean number of concomitant comorbidities was 3.0 (SD 2.4). There were no statistically significant differences in patient demographics between those who attended early follow-up (103) and those who did not (37). Anti-CCP was not collected consistently due to lack of universal funding coverage for test, and thus is not reported.

**Table 1:** Patient demographics of entire cohort, those with 6-week follow-up and those without

Variables	All Patients	6 week follow up visit	No 6 week follow up visit	*p* value
	Mean Values (SD)			
N	140	103	37	
Female, no. (%)	86 (61.4)	64 (62.1)	22 (59.5)	0.96
Age, mean (SD) (years)	55.78 (15.69)	56.23 (15.98)	54.54 (13.00)	0.86
Meets 2010 ACR/EULAR Criteria, no. (%)	113 (80.7)	85 (82.5)	28 (75.7)	0.90
Mean symptom duration (days) at first visit, mean (SD)	160.24 (107.37)	158.76 (105.48)	164.35 (114.56)	0.96
Rheumatoid Factor positive, no. (%)	156 (58.6)	58 (58.6)	20 (58.8)	0.77
Smoking Status, no. (%)				
Current smoker	19 (13.6)	14 (13.6)	5 (13.5)	0.70
Ex-smoker	64 (45.7)	45 (43.7)	19 (51.4)	
Never	57 (40.7)	44 (42.7)	13 (35.1)	
Caucasian, no. (%)	117 (83.6)	87 (84.5)	30 (81.1)	0.85
Marital status, no. (%)				
Single	22 (15.7)	18 (17.5)	4 (10.8)	1.00
Common Law	6 (4.3)	5 (4.9)	1 (2.7)	
Married	91 (65.0)	63 (61.2)	28 (75.7)	
Education, no. (%)				
Elementary school	6 (4.3)	5 (4.9)	1 (2.7)	0.92
High school	56 (40.0)	40 (38.8)	16 (43.2)	
College/Trade school	41 (29.3)	27 (26.2)	14 (37.8)	
University Bachelor	25 (17.9)	22 (21.4)	3 (8.1)	
Masters	7 (5.0)	6 (5.8)	1 (2.7)	
PhD	5 (3.6)	3 (2.9)	2 (5.4)	
Income, no. (%)				
None	6 (4.3)	5 (4.9)	1 (2.7)	0.80
< $20,000	21 (15.0)	17 (16.5)	4 (10.8)	
$20,000–$50,000	46 (32.9)	28 (27.2)	18 (48.6)	
$50,000–$100,000	36 (25.7)	28 (27.2)	8 (21.6)	
> $100,000	10 (7.1)	8 (7.8)	2 (5.4)	
Do Not Wish to Answer	21 (15.0)	17 (16.5)	4 (10.8)	
Employment, no. (%)				
Employed	69 (49.3)	51 (49.5)	18 (48.6)	0.66
Retired	43 (30.7)	31 (30.1)	12 (32.4)	
Homemaker	10 (7.1)	6 (5.8)	4 (10.8)	
Student	6 (4.3)	5 (4.9)	1 (2.7)	
Disabled	4 (2.9)	2 (1.9)	2 (5.4)	
Sick Leave	3 (2.1)	3 (2.9)	0 (0)	
Maternity Leave	1 (0.7)	1 (1.0)	0 (0)	
Unemployed	4 (2.9)	4 (3.9)	0 (0)	
Number comorbidities present, mean (SD)	3.03 (2.32)	2.99 (2.38)	3.13 (2.21)	0.95

### Remission and low disease-activity state

The proportion of patients in newly achieving either remission or low disease-activity state as per CDAI, SDAI and DAS28 scores are shown in Table 2. At baseline, 66 % of patients were classified as CDAI high disease activity state, with 9 % in LDA state. By 6 weeks, 17 % had achieved CDAI remission with an additional 1 % achieving CDAI remission by 12 weeks; there were a larger proportion of patients entering into early CDAI remission (16 %) versus later remission (1 %) (*p* < 0.0002). Similar results were found for DAS28 remission (Table 2). By 6 weeks, 59 % had achieved either DAS28 remission or low disease activity state, with 74 % achieving either state by 12 weeks. There were a larger proportion of patients that achieved DAS28 remission in the early vs. late periods (*p* < 0.00002).

**Table 2:** The proportion of patients in CDAI/SDAI/DAS28 remission, low activity, moderate activity and high activity states at 0, 6 and 12 weeks. CDAI: Clinical Disease Activity Index; SDAI: Simplified Disease Activity Index; DAS28: Disease Activity Score in 28 joints (using ESR)

Remission score		Baseline No. (%)	6 weeks No. (%)	12 weeks No. (%)	Early (0–6 weeks) vs. Late (6–12 weeks) (p)
CDAI	N	102	96	89	
	Remission ( ≤ 2.8)	0 (0.0)	16 (16.7)	17 (19.1)	0.0002
	Low Activity (2.9–10.0)	9 (8.8)	25 (26.0)	48 (53.9)	0.15
	Moderate Activity (10.1–22.0)	26 (25.5)	37 (38.5)	21 (23.6)	
	High Activity ( > 22)	67 (65.7)	18 (18.8)	3 (3.4)	
SDAI	N	97	58	37	
	Remission ( ≤ 3.3)	0 (0.0)	4 (6.9)	3 (8.1)	
	Low Activity (3.4–11.0)	3 (3.1)	13 (22.4)	16 (43.2)	0.08
	Moderate Activity (11.1–26)	15 (15.5)	19 (32.8)	13 (35.1)	
	High Activity ( ≥ 26)	79 (81.4)	22 (37.9)	5 (13.5)	
DAS28	N	94	85	76	
	Remission ( ≤ 2.4)	2 (2.1)	22 (25.9)	23 (30.3)	0.00002
	Low Activity (2.5–3.6)	9 (9.6)	28 (32.9)	33 (43.4)	0.41
	Moderate Activity (3.7–5.5)	40 (42.6)	29 (34.1)	19 (25.0)	
	High Activity ( ≥ 5.5)	43 (45.7)	6 (7.1)	1 (1.3)	

### Disease activity at early follow-up

Mean change in disease activity scores at each appointment are presented in Additional file 1. Every disease activity score differed across time points. Between baseline and 6 weeks, there was a significant difference in mean score for all measures except ESR. Between 6 and 12 weeks, there was a significant difference for all measures except physician global assessment (*p* = 0.052), CRP (*p* = 0.37), DAS28ESR (*p* = 0.292), and SDAI (*p* = 0.17). Between baseline and 12 weeks, there was a significant change in mean scores for all outcomes. Compared to the later period (6–12 weeks), there was a significantly greater early improvement for SJC, SJC28, pain, fatigue, sleep, patient global assessment, physician global assessment, CRP, DAS28ESR, DAS28CRP, CDAI and SDAI.

### Methotrexate monotherapy vs. combination DMARD therapy

Of 103 patients included, 94 patients were on methotrexate monotherapy and only 9 patients were on combination therapy with hydroxychloroquine. Within monotherapy or combination therapy groups, all disease activity measures significantly differed at each time point (p ≤ 0.001 for TJC, SJC, TJC28, SJC, pain, fatigue, sleep, patient global assessment, HAQ, physician global assessment, CRP, DAS28ESR, DAS28CRP, CDAI, SDAI; *p* < 0.02 for ESR). Patients treated with MTX monotherapy demonstrated a significantly greater early improvement for SJC, SJC28, pain, fatigue, sleep, PTGA, HAQ, MDGA, DAS28ESR, DAS28CRP, CDAI, and SDAI (all p ≤ 0.001). In the small cohort of patients on combination with HCQ (*n* = 9), there was a non-significant trend toward greater early improvement.

### Concomitant steroid use

Of the 103 patients included, 22 patients did not receive steroids, 15 received oral steroids, 34 received intra-articular steroids only, 13 received intra-muscular steroids only, and 19 patients received combination steroids of at least two routes. Mean disease activity scores at each time point did not significantly differ between steroid groups. The patient group treated with intra-articular steroids alone yielded the highest number of disease measures that demonstrated significant early improvement. Within the small cohort treated with combination steroids (*n* = 19) there was significant early improvement for only two disease measures- fatigue (*p* < 0.001) and PTGA (*p* < 0.001) but this group was likely underpowered. In the cohort not given steroids, there was significant early improvement for TJC and PTGA (*p* < 0.03), as well as MDGA, DAS28ESR and CDAI (all *p* < 0.02).

### Side effects and tolerability

There were no dropouts secondary to methotrexate intolerance over the first 3 months. Few reported headache (3 %), nausea (4 %), and gastrointestinal symptoms (1 %).

## Discussion

Our study demonstrated an early effect of optimal dosing of scMTX in patients with early RA, with significantly larger early improvement in several disease activity scores and higher proportion achieving early CDAI and DAS28 remission.. Use of concomitant intra-articular steroid yielded the highest number of disease activity scores with significant early improvement. The small cohort of patients on combination MTX and HCQ trended toward a greater degree of early improvement, however statistical significance was not achieved - likely secondary to small sample size. Unlike some practices, we often initiate optimal dosing of MTX at the onset of treatment instead of escalating over time. This could contribute to the rapid kinetics of MTX.

Previous data published on MTX therapy in patients with early rheumatoid arthritis have typically used 12 weeks as the initial follow-up point [7]. In this study, the kinetics of sc MTX are more rapid. Many of the patients received concomitant steroids which may facilitate a rapid response, but do not necessarily contribute to the sustained response. Although patients receiving either oral, intramuscular or intra-articular steroids had a higher number of disease activity measures that demonstrated significant early improvement, the cohort of patients who did not receive steroids still demonstrated significant early improvement for five disease activity measures.

Although all patients were from a single centre, baseline characteristics were similar for patients who had 6 weeks visit versus those who did not. This site had baseline demographic data that were not different from the overall CATCH cohort [2]. Assessing patients from a single centre allowed for consistent education, disease scoring and follow-up protocols. There were few patients lost to follow-up and little missing data, avoiding selection bias.

The use of combination therapy with HCQ was also assessed as another potential confounder. Patients who initially received combination therapy had significantly higher disease activity scores at each time point, as compared to those receiving MTX monotherapy, suggesting that patients in the combination group had more active RA at presentation. No disease activity measures within the combination MTX + HCQ group demonstrated a more significant improvement in the early period compared to the later period, likely due to the small N. We could not study oral MTX as it was too rarely used in this practice and there was no triple therapy. The lack of a control group is problematic for the interpretation of the results (po MTX), lack of randomization and prescribing biases of steroid use. The relatively low DAS28 in the ERA incident cohort at onset would tend to give less of a change in DAS28 compared to starting at higher disease activity. Despite these limitations, there is no doubt that even when stratifying by steroid use, there is an early rapid response in those starting optimal doses of sc MTX. A sub-analysis from the TEAR trial showed that even with MTX monotherapy or triple therapy, response at 12 weeks was predictive of future low disease activity, but this study did not look at 6 weeks response [16]. Certainly MTX seems to have a larger effect early (first 6 weeks) compared to the 2^nd^ 6 weeks so perhaps future response can be predicted even earlier with optimal dosing of sc MTX.

## Conclusions

We found that optimal initial dosing with sc MTX in patients with early RA was well-tolerated and yielded rapid (within 6 weeks) significant improvement in many disease measures, including achieving remission and low disease activity state. The use of intra-articular steroids enhanced rapid clinical improvement, as compared to use of steroids via other routes. Future multicenter studies are needed to further assess the rapid kinetics of MTX therapy.

## Additional file

Additional file 1: Comparison of mean change in outcome measurement scores between early period (0–6 weeks) and late period (6–12 weeks). DAS28ESR: Disease Activity Score in 28 joints (using ESR); TJC28: Tender Joint Count in 28 joints; SJC28: Swollen Joint Count in 28 joints; PTGA: Patient Global Assessment; HAQ: Health Assessment Questionnaire Disability Index; MDGA: Physician Global Assessment; ESR: Erythrocyte Sedimentation Rate; CRP: C-Reactive Protein; DAS28CRP: Disease Activity Score in 28 joints (using CRP); CDAI: Clinical Disease Activity Index; SDAI: Simplified Disease Activity Index. (PDF 84 kb)

## Abbreviations

ACR: American College of Rheumatology
ANOVA: Analysis of variance
CATCH: Canadian early arthritis cohort
CDAI: Clinical disease activity index
DAS28: Disease activity score (28 joint count)
DMARD: Disease modifying anti-rheumatic drug
EIA: Early inflammatory arthritis
HCQ: Hydroxychloroquine
MTX: Methotexate
RA: Rheumatoid arthritis
sc: Subcutaneous
scMTX: Subcutaneous methotrexate
SDAI: Simplified disease activity index
