# Supplementary material for: The rapid kinetics of optimal treatment with subcutaneous methotrexate in early inflammatory arthritis: an observational study
Source: BMC Musculoskelet Disord. 2016 Aug 24;17(1):364. doi: 10.1186/s12891-016-1213-6 (PMC4997744; doi:10.1186/s12891-016-1213-6)
Supplement: Additional file 1: — Comparison of mean change in outcome measurement scores between early period (0–6 weeks) and late period (6–12 weeks). DAS28ESR: Disease Activity Score in 28 joints (using ESR); TJC28: Tender Joint Count in 28 joints; SJC28: Swollen Joint Count in 28 joints; PTGA: Patient Global Assessment; HAQ: Health Assessment Questionnaire Disability Index; MDGA: Physician Global Assessment; ESR: Erythrocyte Sedimentation Rate; CRP: C-Reactive Protein; DAS28CRP: Disease Activity Score in 28 joints (using CRP); CDAI: Clinical Disease Activity Index; SDAI: Simplified Disease Activity Index. (PDF 84 kb) [file 12891_2016_1213_MOESM1_ESM.pdf]

Additional File 1: Comparison of mean change in outcome measurement scores between early period (0 – 6 weeks) and late period (6 – 12 weeks). DAS28ESR: Disease Activity Score in 28 joints (using ESR); TJC28: Tender Joint Count in 28 joints; SJC28: Swollen Joint Count in 28 joints; PTGA: Patient Global Assessment; HAQ: Health Assessment Questionnaire Disability Index; MDGA: Physician Global Assessment; ESR: Erythrocyte Sedimentation Rate; CRP: C-Reactive Protein; DAS28CRP: Disease Activity Score in 28 joints (using CRP); CDAI: Clinical Disease Activity Index; SDAI: Simplified Disease Activity Index.

**Change (Δ) in Outcome**	**Cohort**	**N**	**Early Period (0–6 wks)**	**Late Period (6-12 wks)**	**Mean Difference Early vs. Late**	**p**
ΔDAS28ESR	Entire cohort	72	-1.9 (0.4)	-0.2 (0.3)	-1.7 (2.2)	0.00**
	Monotherapy	66	-1.8 (1.3)	-0.2 (1.1)	-1.7 (2.1)	0.00**
	MTX+HCQ	6	-2.6 (2.4)	-0.9 (1.4)	-1.8 (3.7)	0.3
	No Steroid	17	1.7 (1.1)	-0.6 (1.0)	-1.1 (1.8)	0.02*
	PO Steroid	10	-2.0 (1.7)	0.3 (1.0)	-2.3 (2.3)	0.01*
	IA Steroid	22	-2.2 (1.3)	-0.4 (1.3)	-1.8 (2.4)	0.00**
	IM Steroid	9	-1.7 (1.2)	0.5 (1.4)	-2.2 (2.3)	0.02*
	Steroid Combination	14	-1.7 (1.6)	-0.4 (1.0)	-1.3 (2.4)	0.06

ΔTJC28	Entire cohort	86	-2.6 (1.9)	-3.0 (1.3)	0.4 (10.4)	0.7
	Monotherapy	78	-1.8 (6.5)	-3.1 (4.9)	1.3 (9.9)	0.3
	MTX+HCQ	8	-9.9 (9.6)	-1.6 (3.3)	-8.3 (12.1)	0.1
	No Steroid	17	-2.0 (3.9)	-2.9 (2.7)	0.9 (5.5)	0.5
	PO Steroid	12	-3.6 (6.8)	-2.5 (4.2)	-1.1 (9.3)	0.7
	IA Steroid	28	-4.1 (8.8)	-2.9 (5.1)	-1.2 (12.4)	0.6
	IM Steroid	12	0.08 (5.1)	-1.4 (4.9)	1.5 (9.0)	0.6
	Steroid Combination	17	-1.7 (8.2)	-4.7 (6.0)	2.9 (12.7)	0.4
ΔSJC28	Entire cohort	86	-5.9 (1.6)	-1.4 (0.9)	-4.5 (7.7)	0.00**
	Monotherapy	76	-5.6 (5.7)	-1.3 (3.4)	-4.4 (7.6)	0.00**
	MTX+HCQ	7	-8.1 (8.8)	-2.0 (2.6)	-6.1 (10.3)	0.1
	No Steroid	17	-3.6 (4.0)	-2.2 (1.6)	-1.4 (4.3)	0.2
	PO Steroid	12	-8.0 (6.5)	0.00 (1.2)	-8.0 (6.7)	0.00**
	IA Steroid	28	-7.8 (6.8)	-1.6 (3.3)	-6.2 (8.2)	0.00**
	IM Steroid	12	-7.4 (5.9)	1.7 (2.8)	-9.1 (7.9)	0.00*
	Steroid Combination	17	-2.8 (4.0)	-3.2 (4.2)	0.4 (6.6)	0.8
ΔPain	Entire cohort	86	-3.4 (1.9)	-0.01 (0.75)	-3.44 (5.8)	0.00**
	Monotherapy	77	-3.3 (3.6)	0.03 (2.9)	-3.3 (5.9)	0.00**

	MTX+HCQ	9	-3.8 (3.9)	-0.4 (1.2)	-3.4 (4.8)	0.07
	No Steroid	18	-3.1 (3.8)	-0.5 (3.6)	-2.6 (6.7)	0.1
	PO Steroid	13	-1.7 (4.3)	-0.6 (3.3)	-1.1 (7.0)	0.6
	IA Steroid	27	-4.5 (3.3)	0.4 (2.5)	-4.9 (4.9)	0.00**
	IM Steroid	12	-3.1 (3.4)	1.1 (2.3)	-4.3 (5.2)	0.02*
	Steroid Combination	16	-3.2 (3.4)	-0.6 (2.4)	-2.7 (5.2)	0.06
ΔFatigue	Entire cohort	86	-2.0 (0.8)	-0.2 (0.7)	-1.8 (5.0)	0.001**
	Monotherapy	77	-1.9 (3.1)	-0.2 (2.7)	-1.8 (5.0)	0.003**
	MTX+HCQ	9	-2.2 (4.0)	-0.4 (1.3)	-2.3 (5.3)	0.2
	No Steroid	18	-1.0 (3.6)	-1.6 (3.1)	0.6 (5.9)	0.7
	PO Steroid	13	-1.7 (3.6)	-0.2 (3.5)	-1.6 (6.3)	0.4
	IA Steroid	25	-2.7 (3.4)	-0.4 (2.4)	-2.3 (5.0)	0.02*
	IM Steroid	12	-1.8 (2.7)	0.8 (1.3)	-2.6 (3.4)	0.02*
	Steroid Combination	14	-2.2 (2.2)	1.1 91.7)	-3.3 (3.0)	0.00**
ΔSleep	Entire cohort	82	-2.7 (0.9)	-0.003 (1.3)	-2.6 (5.0)	0.00**
	Monotherapy	75	-2.6 (3.4)	-0.007 (2.7)	-2.6 (5.1)	0.00**
	MTX+HCQ	7	-3.2 (3.4)	-0.4 (1.3)	-2.7 (4.4)	0.2
	No Steroid	18	-2.3 (3.6)	-0.1 (3.3)	-2.2 (6.0)	0.1
	PO Steroid	13	-2.1 (3.6)	0.8 (1.9)	-2.9 (4.5)	0.04*

	IA Steroid	25	-3.2 (4.0)	-0.4 (3.0)	-2.8 (5.8)	0.02*
	IM Steroid	12	-2.9 (2.5)	0.7 (1.9)	-3.6 (4.0)	0.01**
	Steroid Combination	14	-2.4 (2.8)	-0.8 (1.5)	-1.7 (3.3)	0.08
ΔPTGA	Entire cohort	86	-3.8 (1.9)	0.15 (0.8)	-3.9 (5.8)	0.00**
	Monotherapy	77	-3.8 (3.5)	0.3 (2.9)	-4.08 (5.70)	0.00**
	MTX+HCQ	9	-3.8 (4.4)	-1.2 (2.8)	-2.7 (6.8)	0.3
	No Steroid	18	-3.8 (3.6)	-0.6 (2.6)	-3.3 (5.6)	0.03*
	PO Steroid	13	-2.7 (4.7)	-0.2 (4.0)	-2.5 (8.1)	0.3
	IA Steroid	27	-4.1 (3.4)	0.2 (3.3)	-4.3 (5.9)	0.00**
	IM Steroid	12	-3.3 (2.6)	1.2 (1.6)	-4.5 (3.9)	0.00**
	Steroid Combination	16	-4.5 (3.3)	0.5 (2.7)	-4.9 (5.1)	0.00**
ΔHAQ	Entire cohort	73	-0.4 (0.2)	-0.3 (0.1)	-0.2 (0.9)	0.1
	Monotherapy	66	-0.5 (0.6)	-0.3 (0.4)	-0.2 (0.8)	0.05*
	MTX+HCQ	7	-0.2 (1.2)	-0.4 (0.06)	0.2 (1.7)	0.8
	No Steroid	16	-0.3 (0.7)	0.4 (0.5)	0.1 (0.9)	0.7
	PO Steroid	10	-0.5 (0.5)	-0.2 (0.3)	-0.3 (0.8)	0.2
	IA Steroid	23	-0.5 (0.8)	-0.3 (0.6)	-0.2 (1.2)	0.4
	IM Steroid	12	0.5 (0.6)	-0.09 (0.3)	-0.4 (0.8)	0.1
	Steroid	12	0.4 (0.6)	-0.3 (0.3)	-0.09 (0.7)	0.7

	Combination					
ΔMD GA	Entire cohort	82	-3.1 (0.7)	-0.6 (0.6)	-2.5 (3.9)	0.00**
	Monotherapy	74	-3.0 (2.7)	-0.5 (2.0)	-2.5 (3.9)	0.00**
	MTX+HCQ	8	-4.1 (2.7)	-0.9 (1.9)	-3.2 (4.0)	0.06
	No Steroid	17	2.5 (1.8)	-0.6 (2.1)	-1.9 (3.0)	0.02*
	PO Steroid	11	-3.5 (2.5)	0.3 (1.2)	-3.8 (2.6)	0.00**
	IA Steroid	26	-3.4 (3.2)	-0.9 (1.9)	-2.6 (4.4)	0..01*
	IM Steroid	12	-3.5 (2.6)	0.3 (1.4)	-3.8 (3.6)	0.00**
	Steroid Combination	16	-2.6 (3.0)	-1.2 (2.8)	-1.4 (4.7)	0.3
ΔESR	Entire cohort	76	-9.0 (10.5)	-3.8 (9.1)	-5.2 (66.5)	0.5
	Monotherapy	69	-7.3 (37.1)	-4.6 (33.8)	-2.7 (67.8)	0.8
	MTX+HCQ	7	-25.7 (38.4)	4.6 (13.7)	-30.3 (48.7)	0.2
	No Steroid	17	-10.0 (18.0)	-0.4 (6.6)	-9.6 (20.3)	0.07
	PO Steroid	11	23.4 (76.6)	-21.9 (76.1)	45.3 (152.4)	0.4
	IA Steroid	24	-19.7 (26.2)	-0.5 (19.4)	-19.2 (34.6)	0.01*
	IM Steroid	10	-12.4 (13.6)	-1.3 (10.3)	-11.1 (20.4)	0.1
	Steroid Combination	14	-12.3 (26.1)	-0.9 (23.2)	-11.4 (46.0)	0.4
ΔCRP	Entire cohort	33	-13.8 (7.6)	-4.4 (7.0)	-9.38 (24.5)	0.04*

	Monotherapy	29	-11.1 (15.2)	-4.3 (16.7)	-6.8 (24.0)	0.1
	MTX+HCQ	4	-33.3 (20.1)	-4.95 (10.0)	-28.3 (22.7)	0.09
	No Steroid	5	-14.8 (19.8)	-4.0 (9.0)	-10.8 (16.5)	0.2
	PO Steroid	4	-16.5 (28.5)	3.0 (5.4)	-19.5 (26.9)	0.2
	IA Steroid	14	-14.3 (17.2)	-2.8 (6.3)	-11.5 (19.2)	0.04*
	IM Steroid	4	-19.5 (18.8)	-0.5 (1.0)	-18.0 (18.9)	0.2
	Steroid Combination	6	-6.6 (6.2)	-16.0 (34.8)	9.3 (38.2)	0.6
ΔDAS 28CRP	Entire cohort	32	-2.3 (0.7)	-0.2 (0.5)	-2.2 (2.3)	0.00**
	Monotherapy	28	-2.2 (1.2)	-0.06 (1.1)	-2.1 (1.9)	0.00**
	MTX+HCQ	4	-3.0 (3.2)	-0.9 (1.5)	-2.1 (4.7)	0.4
	No Steroid	5	-1.5 (1.2)	-0.9 (1.2)	-0.6 (2.4)	0.6
	PO Steroid	4	-3.8 (1.6)	0.7 (1.0)	-4.5 (1.8)	0.02*
	IA Steroid	13	-2.6 (1.5)	-0.3 (1.2)	-2.6 (2.2)	0.00**
	IM Steroid	4	-1.6 (1.2)	0.5 (1.2)	-2.1 (2.2)	0.2
	Steroid Combination	6	-1.8 (1.5)	-0.8 (0.5)	-1.0 (1.9)	0.3
ΔCDAI	Entire cohort	82	15.3 (3.7)	-4.6 (2.7)	-10.9 (20.1)	0.00**
	Monotherapy	74	-14.2 (12.0)	-4.5 (10.3)	-9.7 (18.9)	0.00**
	MTX+HCQ	8	-26.0 (22.8)	-5.8 (8.3)	-20.2 (29.2)	0.09
	No Steroid	17	-12.4 (7.7)	-6.0 (5.8)	-6.4 (10.5)	0.02*

	PO Steroid	11	-17.7 (17.0)	-2.0 (7.0)	-15.8 (20.0)	0.02*
	IA Steroid	26	-18.5 (16.0)	-5.6 (11.1)	-12.8 (23.1)	0.01**
	IM Steroid	12	-14.4 (11.5)	2.0 (8.3)	-16.4 (17.9)	0.1
	Steroid Combination	16	-12.5 (13.9)	-8.5 (12.7)	-4.0 (23.8)	0.5
ΔSDAI	Entire cohort	31	-33.6 (11.6)	-6.9 (8.7)	-26.8 (36.4)	0.00**
	Monotherapy	27	-29.9 (20.5)	-6.0 (19.8)	-23.8 (33.3)	0.001**
	MTX+HCQ	4	-59.0 (42.7)	-12.5 (14.6)	-46.5 (55.6)	0.19
	No Steroid	5	-12.4 (12.5)	-11.1 (12.9)	-15.3 (16.0)	0.1
	PO Steroid	4	-51.4 (38.7)	5.0 (6.8)	-56.4 (36.4)	0.05*
	IA Steroid	12	-37.4 (26.4)	-2.5 (11.2)	-35.0 (30.3)	0.00**
	IM Steroid	4	-32.8 (24.9)	1.5 (11.5)	-34.4 (33.4)	0.1
	Steroid Combination	6	-20.7 (20.0)	-25.5 (32.0)	4.8 (45.1)	0.8
